# Anticipating El Niño–related health risks in Latin America and the Caribbean: from climate information to public health preparedness

**DOI:** 10.3389/fpubh.2026.1959622

**Published:** 2026-10-05

**Authors:** Esteban Ortiz-Prado, Jorge Vasconez-Gonzalez, Rasa Zalakeviciute, Juan S. Izquierdo-Condoy

**Affiliations:** 1 One Health Research Group, Universidad de Las Américas, Quito, Ecuador; 2 Biodiversidad, Medio Ambiente y Salud (BIOMAS), Universidad de Las Américas, Quito, Ecuador

**Keywords:** drought, El Niño–southern Oscillation, ENSO, flooding, public health preparedness, vector-borne diseases

## Abstract

El Niño is a recurrent mode of tropical Pacific climate variability with major public health implications for Latin America and the Caribbean. Its development is partly driven by the Bjerknes feedback, a coupled ocean–atmosphere process in which weakened equatorial trade winds reduce coastal upwelling and allow warm surface waters to shift eastward. Warming of the central and eastern tropical Pacific can reshape regional rainfall and temperature patterns, increasing the likelihood of floods, droughts, wildfires, food-system disruption, and health-service interruption in locations with established ENSO teleconnections. This narrative review synthesizes evidence on the environmental and health pathways through which El Niño may affect populations in Latin America and the Caribbean. We distinguish evidence directly linking health outcomes to ENSO or Coastal El Niño from evidence derived from floods, droughts, wildfires, and other hydrometeorological disasters without formal attribution to ENSO. The latter is used to characterize plausible health pathways and preparedness needs rather than ENSO-specific causal effects. Potential consequences include immediate mortality and injury, waterborne and vector-borne infections, disruption of chronic disease care, food insecurity, undernutrition, toxic exposures, psychological distress, and prolonged health-system burden. The timing and persistence of these effects are context-dependent; preparedness and response priorities are therefore organized by operational needs rather than fixed post-event intervals. As of August 2026, authoritative forecasts indicated that El Niño was present and strengthening, providing a current illustration of why anticipatory preparedness is necessary while not guaranteeing any specific regional impact. Early warning, flood-risk mapping, water, sanitation, and hygiene interventions, epidemiological and entomological surveillance, continuity of health services, and community-centered risk communication should therefore be adapted to local forecasts, vulnerabilities, and surveillance data.

## 1 Introduction

El Niño emerges from the positive Bjerknes feedback: the weakening of the trade winds allows warmer western Pacific waters to shift eastward, increasing sea-surface temperatures along the Peru–Ecuador coast and enhancing atmospheric moisture (1, 2). This process promotes large-scale convection and intense rainfall over the eastern equatorial Pacific (3–5). As convection and rainfall shift toward the coasts of Peru and Ecuador, El Niño also disrupts regional and global precipitation patterns (6). Under anthropogenic climate change, the Intergovernmental Panel on Climate Change (IPCC) has projected that ENSO-related precipitation variability is very likely to increase, suggesting that these hydroclimatic dynamics may become more intense and disruptive (7).

El Niño intensity is commonly classified using the three-month running mean of the Oceanic Niño Index (ONI), based on sea-surface temperature anomalies in the Niño 3.4 region in the central equatorial Pacific Ocean (8). Operational thresholds usually define events as weak (+0.5 to +0.9 °C), moderate (+1.0 to +1.4 °C), and strong (≥ + 1.5 °C) (Table 1) (9). Basin-scale ENSO and Coastal El Niño should not be treated as interchangeable exposures. Basin-scale ENSO is monitored in the central equatorial Pacific, particularly the Niño 3.4 region, whereas Coastal El Niño refers to anomalous warming concentrated in the far eastern Pacific, especially the Niño 1 + 2 region adjacent to Peru and Ecuador. Coastal events can produce major regional impacts even when the basin-scale ENSO state is neutral or comparatively weak. Throughout this review, the term “Coastal El Niño” is therefore used only when supported by regional classification or Niño 1 + 2 conditions.

**Table 1:** Conventional ONI-based El Niño intensity categories.

El Niño severity	Precipitation and flooding	Drought and hydrology	Agricultural and socioeconomic consequences
Weak (+0.5 to +0.9 °C)	Mild anomalies; slightly increased or near-normal rainfall; isolated minor flooding in northern Peru and coastal Ecuador (22, 122)	Mild rainfall deficit in sensitive areas (e.g., Amazon, Central American Dry Corridor, Andes Sierra); river flows close to average (123–125)	Low agricultural and economic impact; mild irrigation constraints; minor infrastructure damage
Moderate (+1.0 to +1.4 °C)	Increased rainfall in typical high-risk regions, with moderate flooding in selected basins in northern Peru and coastal Ecuador (126, 127)	Moderate drought in arid regions, including northern Brazil and the Central American Dry Corridor; moderately altered river flows (128–130)	Moderate agricultural losses; food insecurity, hydroelectricity crisis possible focal outbreaks; intermediate mitigation costs, lives and money loss
Strong (+1.5 to +1.9 °C)	Torrential rainfall and extensive coastal flooding, including extreme anomalies in northern Peru and coastal Ecuador (131)	Intense drought in the Amazon region, Andean highland and dryland areas; mountain rivers may reach extreme flood levels (132, 133)	Major agricultural losses; substantial infrastructure damage; increased hospitalizations and disease outbreaks (134–136)
Very strong (≥ + 2.0 °C)	Catastrophic flooding and major hydrological disruption (12, 137, 138)	Extreme drought in critical areas of Amazon, highland Andes, central American dry corridor; severe depletion of reservoirs, wetlands, and water-storage systems (12, 139, 140)	Collapse of vulnerable sectors; food insecurity; epidemic outbreaks; high social and economic costs (60, 141)

Intensity categories describe the magnitude of sea-surface temperature anomalies in the Niño 3.4 region and do not determine local impacts. Hydrological, agricultural, socioeconomic, and health consequences vary according to event type, seasonality, geography, regional teleconnections, exposure, vulnerability, and preparedness. NOAA transitioned to the Relative Oceanic Niño Index for operational ENSO monitoring in February 2026; Coastal El Niño is assessed separately using conditions in the Niño 1 + 2 region. The examples shown are illustrative and should not be interpreted as forecasts or deterministic consequences (9, 12, 22, 28, 122, 123, 125–127, 130–137).

In practical terms, weak El Niño events may produce limited climate anomalies, whereas moderate events can alter regional rainfall and drought patterns. Strong or very strong events can trigger major hydrometeorological extremes. In Peru, for example, the 1982–1983 and 1997–1998 El Niño events produced approximately 3,000 mm of rainfall between September and May along the northern coast, while severe drought affected the southern highlands (10, 11). In Brazil, strong El Niño events are typically associated with drought in the northeast and anomalous rainfall in the south. In Ecuador, the 2017 coastal El Niño, although short-lived, caused intense flooding over a period of approximately 3 months (12).

As of 13 August 2026, NOAA’s Climate Prediction Center reported that El Niño was present and strengthening, with a greater than 90% probability of a very strong event during the Northern Hemisphere fall and winter of 2026–2027. For October–December 2026, NOAA estimated a 69% probability of a historically strong event, defined as a three-month Relative Oceanic Niño Index (RONI) value of at least +2.5 °C (13). Regional assessments also require separate consideration. On 28 August 2026, Peru’s Multisectoral Commission for the Study of El Niño maintained its Coastal El Niño Alert and estimated a probability of at least 62% of extraordinary Coastal El Niño conditions in the Niño 1 + 2 region from September 2026 through January 2027, while estimating a probability of at least 58% of very strong conditions in the Niño 3.4 region during the same period (14). These probabilities describe the occurrence or intensity of ocean–atmosphere conditions and should not be interpreted as equivalent probabilities of flooding, drought, outbreaks, or other regional impacts. Such impacts remain dependent on event evolution, seasonality, regional teleconnections, and local vulnerability. We therefore use the 2026 event as a current illustration of the value of anticipatory preparedness rather than as a prerequisite for the broader relevance of this review.

Accordingly, this review distinguishes health effects directly associated with ENSO or Coastal El Niño from effects supported primarily by the broader disaster-health literature. This distinction is maintained throughout the synthesis to avoid attributing every flood-, drought-, or wildfire-related outcome directly to ENSO.

## 2 Methods

This article was developed as a structured narrative review rather than a systematic review. The targeted literature search in PubMed/MEDLINE, Scopus, and Web of Science was completed on 1 July 2026 and covered records available from database inception to that date, using combinations of terms related to “El Niño,” “ENSO,” “Coastal El Niño,” “Niño 3.4,” “Niño 1 + 2,” “flood,” “drought,” “wildfire,” “health,” “infectious disease,” “vector-borne disease,” “waterborne disease,” “chronic disease,” “food insecurity,” “malnutrition,” “mental health,” “health systems,” and “public health preparedness.” Searches were conducted in English and Spanish. Reference lists of relevant reviews and primary studies were also examined to identify additional publications. Institutional ENSO forecasts were subsequently updated during manuscript revision to reflect the NOAA/CPC assessment issued on 13 August 2026 and the ENFEN assessment issued on 28 August 2026. These contextual updates were separate from the bibliographic search completed on 1 July 2026.

The geographical focus was Latin America and the Caribbean. Studies from other regions were considered when they provided transferable evidence on biological mechanisms, disaster-related health pathways, or public health interventions for which regional evidence was limited. Eligible sources included epidemiological studies, environmental and climate-health studies, evidence syntheses, and institutional guidance addressing ENSO, Coastal El Niño, or health effects associated with floods, droughts, wildfires, and related hydrometeorological hazards. Publications without a clear connection to the environmental, health, or preparedness pathways addressed in this review were excluded.

Institutional reports and guidance were prioritized for the synthesis of preparedness and response measures. Climate forecasts and event-intensity estimates were supported by primary institutional sources, including NOAA’s Climate Prediction Center and ENFEN. Other non-peer-reviewed sources were considered as contextual material and were not treated as ENSO-specific epidemiological evidence.

Evidence was synthesized narratively and classified into four interpretive categories: (1) ENSO-specific evidence, in which exposures were explicitly defined using ENSO indices or identified ENSO events; (2) Coastal El Niño evidence, based on regional warming in the Niño 1 + 2 region or corresponding national definitions; (3) hazard-analog evidence from floods, droughts, wildfires, or other hydrometeorological disasters without formal attribution to ENSO; and (4) operational recommendations derived from institutional guidance or disaster-response practice. No formal risk-of-bias assessment or meta-analysis was undertaken because the objective was interpretive and operational rather than systematic or effect-estimating.

Evidence from outside Latin America and the Caribbean was included only when regional evidence was unavailable or insufficient and when the study informed a general biological, environmental, or health-system pathway. Its transferability is explicitly qualified because baseline epidemiology, infrastructure, climate, and response capacity may differ substantially across settings.

## 3 Environmental impact

### 3.1 Precipitation and flooding

The equatorial Pacific coast, particularly Ecuador and northern Peru, experiences intense rainfall anomalies during strong El Niño events. For example, coastal Piura in Peru received rainfall approximately 30 times higher than normal during the 1997–1998 event (15). In Ecuador, nearly 50% of extreme El Niño-related precipitation anomalies have occurred below 150 meters above sea level, concentrating flood risk in low-lying coastal zones, river basins, and densely populated floodplains (12). These events can produce river overflow, extensive urban and rural flooding, slope instability, and landslides.

By contrast, inland and highland regions may experience drought and sustained warming. Although these areas remain within the tropical belt, high-elevation ecosystems such as páramos and Andean cities are increasingly exposed to temperature shifts that may compound El Niño-related hydrological stress. In Quito, Ecuador, for example, average temperatures increased by 0.95 °C between 2004 and 2024, while minimum temperatures rose by as much as 2.57 °C, illustrating the rapid warming observed in high-altitude tropical urban environments (16). Southern Peru and large areas of the Andean Altiplano, including Bolivia and southern Peru, are often affected by severe rainfall deficits during El Niño (17). Similarly, in Central America, especially the “Dry Corridor,” El Niño can intensify prolonged droughts, affecting water availability, agriculture, and food security (Figure 1) (18, 19).

**Figure 1:**
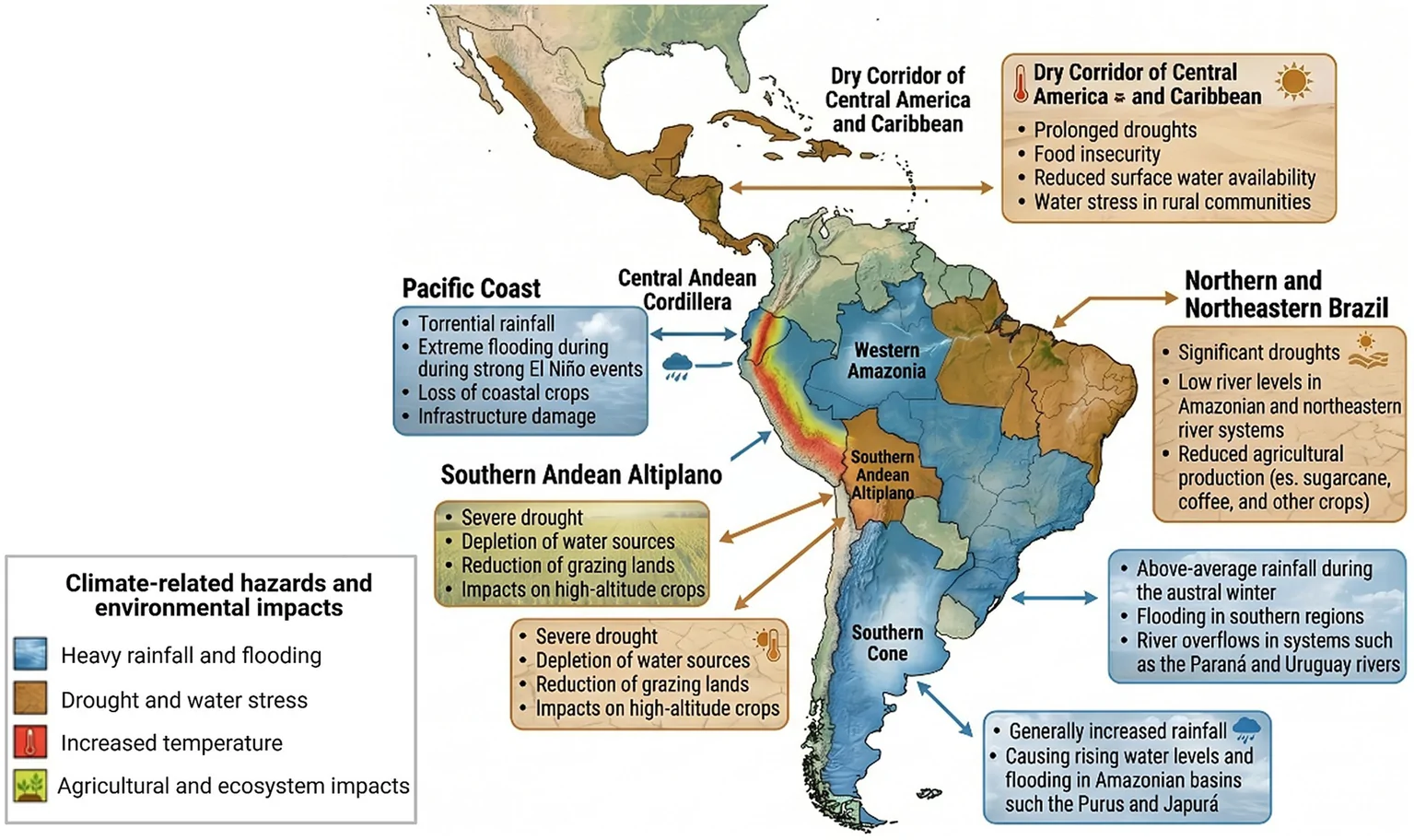
Regional climatic impacts of strong El Niño events across Latin America.

### 3.2 Hydrological extremes and water-security risks

Strong El Niño events can substantially alter hydrological regimes across Latin America. Along the equatorial Pacific coast, intense rainfall can rapidly increase river discharge in coastal basins, particularly in rivers draining the western Andes, producing river overflow, flash flooding, and extensive floodplain inundation (20). In contrast, rainfall-dependent basins in Amazonian and Atlantic-facing regions may experience reduced flows, compromising water availability, agriculture, hydropower generation, and ecosystem function (21). In Ecuador, extreme events can trigger major overflows of the Guayas River and its tributaries, whereas in Peru, water scarcity may intensify in drought-prone southern highland areas (22, 23). These opposing hydrological effects—excess water in coastal basins and water deficits in inland regions—underscore the need for geographically differentiated preparedness strategies (24, 25).

### 3.3 Critical infrastructure failure as a health-risk amplifier

Strong El Niño events frequently damage critical infrastructure, producing cascading failures that substantially amplify public health risks (26). Torrential rainfall, flooding, and landslides can destroy or severely impair transportation networks, water and sanitation systems, power grids, communication infrastructure, and health facilities (27). During the 1997–1998 El Niño, Peru reported damage or destruction to more than 26,000 km of roads, including approximately 4,793 km that were completely destroyed, as well as damage to hundreds of bridges (28). In Ecuador, infrastructure losses were estimated at US$830 million, contributing to an overall economic impact approaching 15% of national GDP (29, 30). Similar patterns were observed during the 2017 Coastal El Niño in Peru, when more than 2,000 km of main roads and hundreds of rural roads were destroyed, 194 bridges collapsed, and irrigation canals and water-supply networks sustained extensive damage (31).

Water supply and sanitation systems are especially vulnerable. Flooding often contaminates or destroys pipelines, treatment plants, and wells; during the 1997–1998 event, 199 water systems serving more than 156,000 people were damaged in Peru alone (32). Health facilities are directly affected as well: approximately 9.5% of all health establishments in Peru (including hospitals) sustained damage, impairing emergency care, routine services, and cold-chain storage of vaccines and medicines (33). Power outages resulting from hydropower system failures disrupt electricity supply, and simultaneous communication breakdowns exacerbate impacts on hospital operations and public health coordination (34, 35).

These infrastructure failures generate profound indirect health consequences. Damaged roads and bridges isolate communities, delaying access to medical care, essential medicines, and even evacuation. Collapsed sanitation systems accelerate outbreaks of waterborne and vector-borne diseases (36). Disruption of electricity and water services exacerbates chronic disease decompensation (e.g., dialysis, insulin refrigeration) and increases mental health strain due to prolonged uncertainty and loss of basic services. In this way, infrastructure damage acts as a critical amplifier of the health burden associated with El Niño (37, 38).

### 3.4 Health risks shaped by hazard, exposure, and vulnerability

The health impact of El Niño–related hydroclimatic extremes depends on three interacting dimensions: hazard, exposure, and vulnerability. Hazard refers to the intensity, duration, and geographic extent of the flood, drought, or associated extreme event; exposure refers to the number and characteristics of people, infrastructure, and services located in affected areas; and vulnerability reflects the capacity of individuals, communities, and health systems to absorb, respond to, and recover from the event (27, 39) The frequency and intensity of extreme precipitation are increasing in many regions (40). Flooding can expose individuals to lead from contaminated soils, deteriorating paint, and dust generated by chipping or peeling paint. Additionally, asbestos present in flood debris can further contribute to air pollution (41). However, the final health burden is strongly shaped by housing quality, settlement in flood-prone areas, river defenses, drainage and sewage systems, early-warning systems, and institutional preparedness (40, 42).

Social vulnerability is central. People with lower socioeconomic status, lower educational attainment, informal employment, limited social support, or precarious housing are more likely to live in high-risk areas and less likely to have resources to evacuate, protect assets, access health care, or recover after the event (40). Individual risk is also shaped by age, sex, disability status, and pre-existing health conditions (43, 44). Women, children, older adults, and people with disabilities are often more likely to experience physical, psychological, and social consequences after floods (40, 45). Young men may have higher immediate mortality, potentially due to risk-taking behaviors, rescue attempts, attempts to cross floodwaters, and fire-related response behaviors (46, 47).

A strong or very strong El Niño in 2026 could therefore expose Ecuador, Peru, and other parts of the Americas to a predictable cascade of health risks. These include direct mortality from drowning and trauma, outbreaks of waterborne and vector-borne diseases, zoonotic infections, interruption of chronic disease care, food insecurity, toxic exposures, and sustained mental health consequences (48).

## 4 Direct and acute health effects

Evidence in this section includes both ENSO-specific observations and hazard-analog studies of floods, landslides, and wildfires. Unless an individual study explicitly identified ENSO as the exposure, the findings should be interpreted as evidence of a plausible post-hazard health pathway rather than as an ENSO-attributable effect.

### 4.1 Drowning and trauma

Drowning is usually the leading cause of immediate mortality associated with floods (49). Historical reviews estimate that approximately 75% of flood-related deaths are attributable to drowning (50). Floods also cause trauma, including falls, wounds, fractures, electrocution, burns, and acute cardiovascular events triggered by stress, physical exertion, or delayed access to emergency care (40).

These immediate effects are particularly relevant in settings with poor drainage systems, informal settlements, limited emergency response capacity, damaged transportation routes, or delayed evacuation (51, 52). In El Niño–affected areas of Ecuador and northern Peru, intense rainfall, river overflow, and landslides may rapidly increase the risk of injury, death, and emergency-care saturation (53, 54).

### 4.2 Impact of wildfires

During El Niño events, global fire emissions increase by between 5.9 and 20.0% (55). Biomass burning from wildfires releases substantial amounts of PM2.5 and trace gases, leading to severe air quality degradation. These particles can penetrate deeply into the respiratory system, causing cardiovascular and respiratory diseases (55). In addition to burns, wildfires also cause psychological trauma, population displacement, disruption of healthcare services, and negative economic impacts (56).

### 4.3 Waterborne and sanitation-related infections

Most evidence relating contaminated floodwater to gastrointestinal and leptospiral infections derives from flood-health studies without formal ENSO attribution. ENSO-specific evidence is available for selected events, including the 2015–2016 El Niño, but remains geographically heterogeneous.

Immediately after flooding, water sources may become contaminated by sewage, solid waste, animal reservoirs, and damaged sanitation infrastructure (57–59). This creates favorable conditions for outbreaks of cholera, typhoid fever, hepatitis A and E, dysentery, leptospirosis, and other forms of acute gastroenteritis (60, 61). For example, Anyamba et al. reported that the 2015–2016 El Niño episode was associated with extreme rainfall and cholera epidemics in Tanzania (60).

Leptospirosis is particularly important after floods because it is linked to exposure to water contaminated with rodent urine, especially in flooded urban and rural environments (62, 63). Risk increases when people walk through floodwater, clean contaminated homes without protection, or live in areas with high rodent density and poor sanitation.

### 4.4 Vector-borne diseases

Evidence linking ENSO to vector-borne disease is based primarily on ecological and time-series associations, whereas evidence following individual floods more broadly supports the biological plausibility of delayed changes in vector abundance and transmission.

Floods and heavy rainfall can increase mosquito breeding sites and modify vector ecology (Figure 2) (64, 65). Increases in dengue, malaria, chikungunya, and other vector-borne diseases have been reported after severe flooding and rainfall anomalies (66–68). Changes in mosquito-borne transmission may follow rainfall or flooding after variable lags, depending on vector ecology, temperature, baseline endemicity, population immunity, and local control measures. No single post-flood interval applies consistently across setting (69, 70).

**Figure 2:**
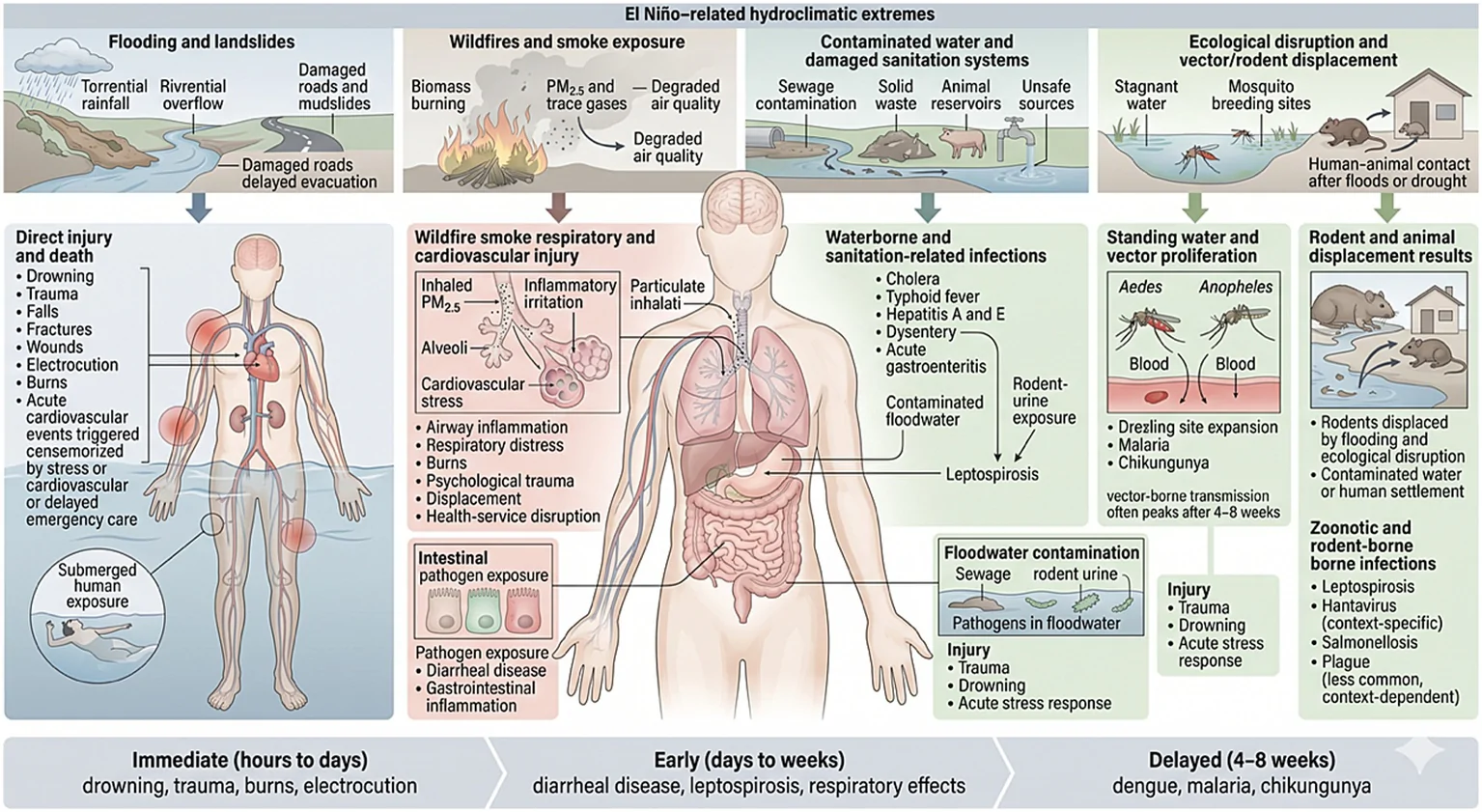
Acute and early health effects of El Niño–related hydroclimatic extremes. Strong El Niño events can trigger multiple, temporally structured health pathways through flooding, landslides, wildfire smoke, damaged sanitation systems, and ecological disruption Immediate effects occurring within hours to days include drowning, trauma, burns, electrocution, and acute cardiovascular events. In the early post-impact period, contaminated water, sewage overflow, and floodwater exposure can increase the risk of diarrheal disease, hepatitis A and E, leptospirosis, and other sanitation-related infections, while wildfire smoke can exacerbate respiratory and cardiovascular conditions. Over subsequent weeks, stagnant water, altered vector ecology, and rodent displacement can amplify transmission of dengue, malaria, chikungunya, leptospirosis, and other zoonotic or vector-borne infections. These pathways illustrate how El Niño-related environmental disruption can rapidly evolve into a cascade of acute clinical events, infectious disease outbreaks, and health-system stress. This figure is a conceptual synthesis combining ENSO-specific evidence and broader disaster-health evidence. Arrows indicate potential pathways rather than established causal effects; temporal phases overlap and do not represent fixed epidemiological intervals.

In Colombia, simultaneous dengue and malaria outbreaks have been observed in association with El Niño–related climate variability, including drought followed by intense rainfall (66). This pattern is especially relevant for the Americas, where El Niño may produce both excess rainfall and drought, depending on the region, creating different ecological conditions for *Aedes* and *Anopheles* transmission (60, 65).

### 4.5 Zoonotic and rodent-borne infections

Except for leptospirosis, evidence for post-disaster increases in rodent-borne and other zoonotic infections is comparatively limited and context-specific; these outcomes should therefore be considered surveillance priorities rather than expected consequences of every El Niño event.

Zoonotic infections may also increase after floods, displacement, ecological disruption, and increased human contact with animal reservoirs (Figure 2) (57, 71). Other infections, including hantavirus, plague, and salmonellosis, have been reported after ecological disruption and disaster-related displacement, although primary evidence remains more limited and varies by pathogen and region (72, 73).

Rodent-borne infections deserve particular attention in areas where flooding displaces rodents into human settlements or where drought and food scarcity modify rodent behavior (74, 75). In this context, leptospirosis has stronger and more consistent evidence after floods, whereas hantavirus should be discussed as an important but more context-specific risk in areas with known reservoirs and compatible ecological conditions.

## 5 Indirect and delayed health effects

### 5.1 Decompensation of chronic diseases

Evidence concerning chronic disease decompensation is derived mainly from the broader disaster-health literature and supports a service-disruption pathway rather than a direct biological effect of ENSO.

Floods disrupt health services, medication supply chains, transportation, dialysis access, refrigeration of insulin and vaccines, and continuity of care (76–78). These disruptions can worsen diabetes, hypertension, chronic obstructive pulmonary disease, cardiovascular disease, cancer, chronic kidney disease, and other long-term conditions (79, 80). Lowe et al. identified interruption of medical treatment and pre-existing comorbidities as important post-flood risk factors (40).

These effects may be amplified when roads, bridges, power grids, and water systems are damaged, limiting access to hospitals, primary care facilities, pharmacies, dialysis centers, vaccination programs, and emergency referral networks (81–83). Patients who depend on continuous medication, refrigerated treatments, oxygen therapy, dialysis, chemotherapy, prenatal care, or regular follow-up are particularly vulnerable (81, 84).

### 5.2 Exposure to contaminants

These pathways are biologically plausible and documented after floods and other disasters, but quantitative evidence directly attributing toxic exposures to ENSO events remains limited.

Floodwaters can mobilize toxic waste, pesticides, fuels, industrial chemicals, sewage, and heavy metals (85, 86). Populations may be exposed through direct skin contact, ingestion, inhalation of contaminated aerosols, or consumption of contaminated food and water. These exposures may increase the risk of acute poisoning, dermatological conditions, respiratory symptoms, gastrointestinal disease, and possible long-term health effects.

In addition, recent drought conditions associated with the last El Niño event of 2023/24 revealed climate-related exposure pathways in urban environments, including air pollutants from increased wildfire events and worsening air and noise pollution linked to extensive diesel-generator use during hydropower shortages and electricity crises in the region (87).

However, quantitative epidemiological evidence remains limited, particularly in Latin American settings. This represents an important research gap, especially in areas where flooding affects agricultural land, mining zones, informal settlements, industrial corridors, fuel storage sites, or waste-disposal areas.

### 5.3 Malnutrition and food insecurity

Floods droughts, landslides, and mudslides associated with strong El Niño events can severely disrupt agricultural production, livestock, fisheries, and food supply chains, leading to sharp increases in food prices, household income losses, and acute food insecurity (Figure 3) (88–91). In coastal regions of Ecuador and northern Peru, torrential rains and flooding may destroy crops, while in the Andean highlands, the Central American Dry Corridor, and northeastern Brazil, prolonged droughts can reduce yields of staple crops such as maize, beans, and rice, as well as forage for livestock (53, 54, 92–94). Additionally, floods affect access to safe drinking water in households, which has been associated with an increased risk of dehydration in children (95).

**Figure 3:**
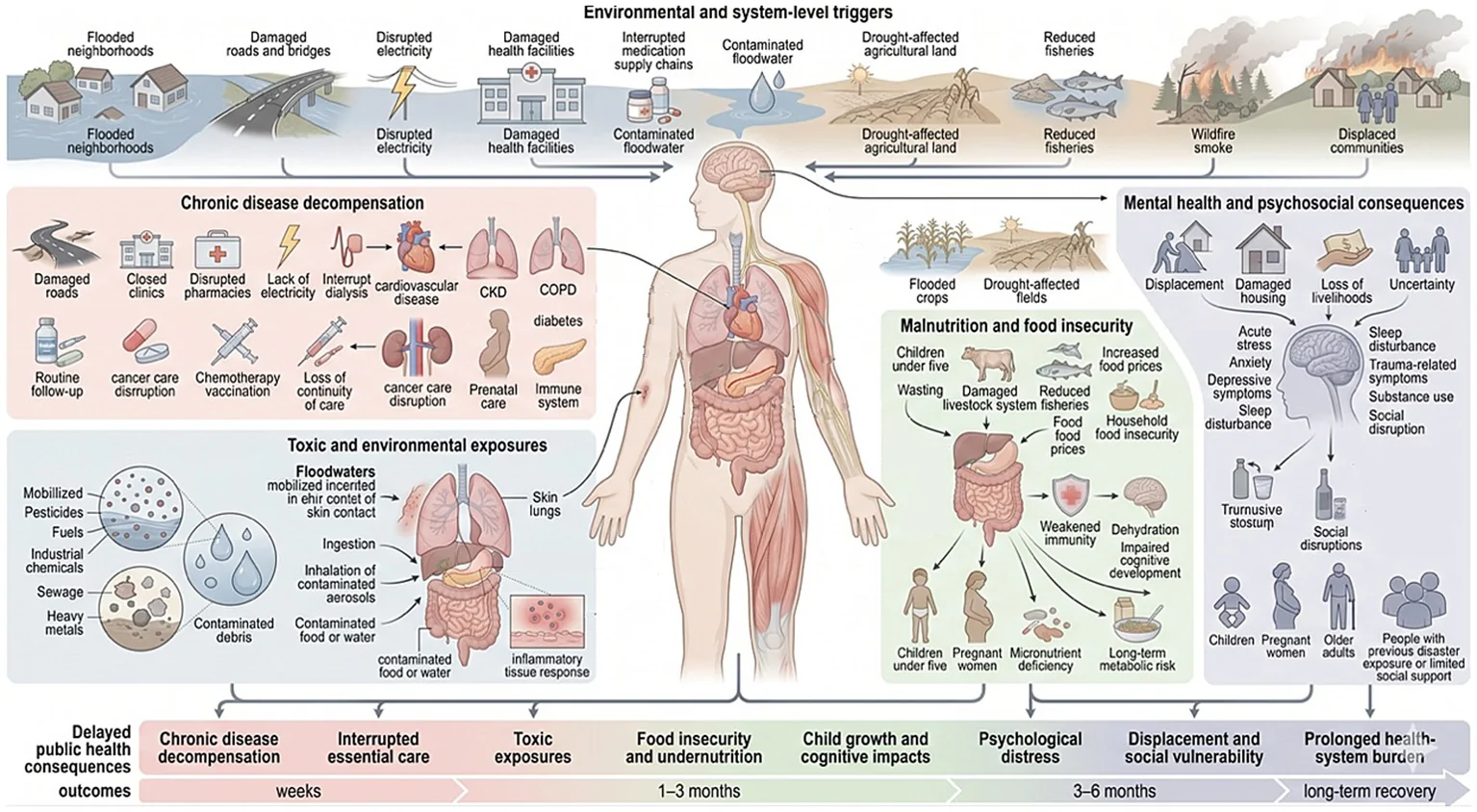
Indirect and delayed health effects of El Niño–related hydroclimatic extremes. El Niño can trigger delayed health impacts through infrastructure damage, disrupted health services, environmental contamination, food-system shocks, and psychosocial stress. These pathways may lead to chronic disease decompensation, toxic exposures, food insecurity and undernutrition, impaired child development, psychological distress, displacement, and prolonged health-system burden over weeks to months. This figure is a conceptual synthesis combining ENSO-specific evidence and broader disaster-health evidence. Arrows indicate potential pathways rather than established causal effects; temporal phases overlap and do not represent fixed epidemiological intervals.

Historical events such as the 1997–1998 El Niño caused massive agricultural losses (53), and the 2023–2024 episode already affected millions: nearly 6.8 million people faced severe food insecurity in Colombia, Ecuador, Peru, and Bolivia, with significant rises in child malnutrition reported in Piura, Tumbes, and Lambayeque in Peru (96).

These disruptions may contribute to nutritional deterioration, particularly among vulnerable populations (96). Children under five, pregnant and lactating women, older adults, and indigenous or rural populations are most affected (97, 98). Acute food insecurity can rapidly lead to wasting, while chronic exposure may contribute to stunting, micronutrient deficiencies, including iron, vitamin A, and zinc deficiency, and increased susceptibility to infections (99, 100).

In Latin America, El Niño–related food insecurity may compound the existing double burden of malnutrition, where undernutrition coexists with overweight and obesity (96, 101, 102). Severe nutritional deterioration is linked to higher child morbidity and mortality from diarrhea, pneumonia, and vaccine-preventable diseases due to weakened immunity (99, 103). Longitudinal evidence also suggests impaired cognitive development, reduced school performance, and lifelong metabolic risks, including higher adult risk of diabetes and cardiovascular disease, in children exposed to early-life nutritional shocks (104, 105).

In the context of 2026, a strong El Niño could exacerbate these effects through combined flooding–drought dynamics, loss of fishing resources, including anchovy in Peru and Ecuador, and rising food prices, potentially affecting millions more. Early nutritional surveillance, distribution of fortified foods, cash or voucher transfers, and protection of agricultural livelihoods are therefore critical components of public health preparedness.

### 5.4 Mental health and psychosocial consequences

Mental-health evidence includes studies of El Niño–related flooding in Peru as well as broader flood-disaster studies. The latter support transferability of the psychosocial pathway but do not establish an ENSO-specific effect size.

Floods, mudslides, wildfires, and associated disasters triggered by strong El Niño events generate profound psychological impacts that often outlast the physical destruction (Figure 3) (106). Affected populations frequently experience acute stress reactions, post-traumatic stress symptoms, generalized anxiety, depressive symptoms, substance use disorders, sleep disturbances, and other forms of psychological distress (107, 108). These conditions arise from direct trauma, loss of life, injury, displacement, material losses, damage to housing and livelihoods, and prolonged uncertainty regarding recovery and basic services (109, 110).

Symptoms typically emerge in the immediate or short-term phase but can persist for months or even years (111). An analysis of the PAMPA cohort following the 2024 floods in Rio Grande do Sul, Brazil, found that greater flood-related burden and displacement were associated with a higher prevalence of moderate-to-severe anxiety and depressive symptoms (112). Similar patterns have been described after major flood disasters, including El Niño–related events in Peru where communities reported elevated rates of anxiety, depression, and post-traumatic stress, particularly in areas with severe flooding and prolonged displacement (113).

Children and adolescents are especially vulnerable (40). Exposure to El Niño–related disasters may increase the risk of post-traumatic stress symptoms, separation anxiety, behavioral problems, developmental disruption, school absenteeism, and long-term mental health sequelae. Pregnant women and mothers may also face heightened risk of perinatal anxiety and depression, which can affect early child development through altered caregiving and household stress (114, 115).

Higher-risk groups overall include women, older adults, people with previous disaster exposure or pre-existing mental health conditions, those who lose housing or livelihoods, indigenous and rural populations, and individuals with limited social support networks (40, 116, 117). Infrastructure damage and food insecurity act as important amplifiers of psychological distress by prolonging exposure to stressors (109, 118).

In the context of a potential strong 2026 El Niño, integrating mental health and psychosocial support into preparedness and response plans is essential. This includes psychological first aid, community-based interventions, school-based programs for children, tele-mental health services, and referral pathways to specialized care. Early intervention may reduce the long-term burden of post-disaster anxiety, depression, trauma-related symptoms, and related disorders.

## 6 Operational priorities across preparedness, response, and recovery

Health risks associated with El Niño–related hydroclimatic hazards do not follow a uniform post-event timetable. Table 2 therefore organizes preparedness and response priorities by operational needs rather than fixed intervals. Emergency care, environmental health protection, continuity of treatment, and nutritional and psychosocial support may be required concurrently. Their activation and duration should be guided by local exposures, surveillance findings, service disruption, and population needs. This author-developed synthesis draws on the evidence and guidance reviewed and has not been validated as a temporal model (48, 67, 81).

**Table 2:** Public health priorities across preparedness, response, and recovery from El Niño–related hydroclimatic hazards.

Operational focus	Health risks and needs	Priority public health actions
Anticipatory preparedness	Exposure to forecast hydroclimatic hazards, social vulnerability, infrastructure fragility, and dependence on essential health services (40, 48)	Integrate climate information with local vulnerability assessments; identify populations requiring assistance; prepare evacuation and communication plans; pre-position essential supplies and establish health-service continuity arrangements (48, 67, 81)
Emergency response during hazardous exposure	Drowning, traumatic injuries, acute illness, displacement, and disruption of emergency care (40, 47, 48)	Activate rescue, triage, emergency treatment, safe shelter, and referral routes; maintain access to essential medicines and care for medically vulnerable individuals (67, 81, 84)
Environmental health protection and infectious-disease surveillance	Unsafe water, damaged sanitation, contaminated environments, and locally relevant waterborne, vector-borne, and zoonotic infections, including leptospirosis (57, 63, 65)	Restore safe water and sanitation; promote hygiene and safe cleanup; investigate suspected outbreaks; adapt epidemiological surveillance and vector control to local transmission patterns and environmental conditions (58, 67, 68, 120, 121)
Continuity and restoration of essential care	Interrupted treatment, chronic disease decompensation, and reduced access to routine and preventive health services (79, 83, 119)	Maintain medication access and safe storage, dialysis, and other essential treatments; restore primary care and referral networks; identify and follow patients whose care has been interrupted (78, 81, 83, 84)
Nutritional and psychosocial support	Food insecurity, nutritional deterioration, psychological distress, and increased needs among displaced or otherwise vulnerable populations (48, 113).	Assess food, nutritional, and psychosocial needs; facilitate access to adequate nutrition; provide appropriate psychosocial support and referral pathways; coordinate health services with community and social support resources (48, 81).
Sustained recovery and follow-up	Persistent physical and psychological morbidity, disability, disrupted chronic care, and continuing social vulnerability (111, 119, 142)	Maintain clinical and psychosocial follow-up; provide rehabilitation according to assessed needs; restore routine services; reassess unmet needs and incorporate lessons learned into future preparedness planning (81, 84)

This table is an author-developed operational synthesis of the evidence and guidance discussed in the review, not a validated temporal classification or an ENSO-specific prediction model. References support the listed risks and actions, rather than fixed onset times or durations. Operational priorities overlap and may need to be implemented concurrently. Their activation and duration should be determined by local forecasts, actual exposures, epidemiological surveillance, service disruption, and population needs. Clinical, nutritional, and psychosocial support may be required from the outset and should not be postponed until a designated recovery phase. Evidence derived from disasters without formal ENSO attribution is used to inform preparedness and response, not to establish ENSO-specific causal effects.

## 7 Prevention and mitigation

The recommendations below differ in evidentiary basis. Safe-water interventions and continuity planning are supported by disaster-health evidence and institutional guidance, whereas some vector-control, zoonotic-surveillance, mobile-care, and psychosocial measures rely more heavily on biological plausibility, operational experience, or context-specific evaluations. They should therefore be adapted to local surveillance and response capacity rather than applied as universally effective interventions.

Reducing the health impact of floods requires anticipatory, coordinated, and risk-based interventions. Priorities include early-warning systems, flood-risk mapping, clearly defined evacuation routes, community drills, and targeted communication strategies (40, 97). These measures are intended to reduce immediate mortality and facilitate timely response, although comparative evidence on the magnitude of their effect remains limited.

Continuity of health services is essential. Hospitals and primary care centers located in vulnerable areas should have contingency plans, protection of critical equipment, backup electricity, alternative logistic routes, and reserves of essential medicines (77, 81, 82). This is particularly important for patients with diabetes, hypertension, chronic kidney disease, cardiovascular disease, respiratory disease, and cancer (83, 84, 119).

Water, sanitation, and hygiene interventions are central to outbreak prevention. Safe water should be guaranteed through chlorination, filters, purification tablets, protected storage, temporary sanitation, cleaning of contaminated wells, and promotion of hand hygiene. Safe-water and hygiene interventions can reduce diarrheal disease risk; their effects on leptospirosis, viral hepatitis, and other post-flood infections are more context-dependent (120, 121).

Epidemiological surveillance should be strengthened from the acute phase through active reporting in health facilities, shelters, and affected communities. Priority syndromes and diseases should include acute diarrhea, fever, respiratory infections, leptospirosis, dengue, malaria, skin infections, and injuries (57, 59, 68). In endemic areas, entomological surveillance is essential to anticipate outbreaks and guide vector-control measures (64, 70).

Where entomological surveillance or local risk assessment indicates increasing vector abundance, vector-control activities should be initiated or intensified before the expected rise in cases (69, 70).

The medical response should combine mobile brigades, temporary care posts, restoration of vaccination services, prenatal care, child health services, provision of continuous medications, and nutritional support. Mental health should be incorporated into the response through psychological first aid, community-based support, and referral pathways for people with persistent or severe symptoms (81, 106, 107).

Risk communication must be clear, practical, and culturally appropriate. Messages should address safe water, hand hygiene, food handling, warning signs, safe evacuation, avoidance of floodwater exposure, prevention of mosquito breeding sites, and early care-seeking. Finally, effective response requires coordination among public health authorities, disaster-risk management agencies, local governments, water and sanitation services, social protection systems, academic institutions, and community organizations (67, 97).

Overall, the available evidence supports preparedness, safe water access, epidemiological surveillance, vector control, continuity of health services, and community-based risk communication as core strategies to reduce the direct and indirect health burden associated with floods (67, 80, 97).

### 7.1 Limitations of the evidence and research gaps

The evidence on the health impact of floods has important limitations. Most studies are retrospective, descriptive, or ecological, which limits causal inference and makes it difficult to evaluate the effectiveness of specific interventions.

There is a shortage of prospective and longitudinal studies examining post-event outcomes such as chronic disease decompensation, nutritional deterioration, mental health disorders, disability, loss of treatment adherence, and long-term health-system disruption (77, 108, 117). In addition, much of the literature comes from high-income or upper-middle-income countries, whereas highly vulnerable regions, including sub-Saharan Africa, South Asia, and rural Latin America, remain underrepresented.

Comparative evidence on specific interventions is also limited. Strategies such as safe-water kits, post-flood fumigation, entomological surveillance, mobile health brigades, and psychosocial support are often recommended based on biological plausibility, operational experience, and institutional guidelines rather than controlled studies (97, 120, 121).

The targeted, non-systematic search and absence of formal risk-of-bias assessment may have introduced selection bias and limit conclusions about the completeness and certainty of the evidence base.

Another limitation is heterogeneity in outcome measurement. Some studies focus only on immediate mortality, whereas others include hospitalizations, outbreaks, outpatient consultations, injuries, mental health outcomes, or service disruption. This lack of standardization limits comparability and may underestimate the true burden of disease.

Future research should integrate climate, hydrological, epidemiological, and social data using harmonized definitions and longitudinal follow-up. This would allow more accurate estimation of the health burden of floods and support more effective anticipatory action in vulnerable settings.

## 8 Conclusion

El Niño–related hydroclimatic extremes can create substantial public health risks in Latin America and the Caribbean through locally variable pathways involving flooding, drought, wildfire smoke, disrupted essential services, food insecurity, and psychosocial stress. ENSO-specific associations should be distinguished from health effects inferred from the broader disaster literature, and neither event intensity nor elapsed time alone determines local outcomes.

Preparedness should integrate climate forecasts with local vulnerability assessments and epidemiological surveillance to guide safe-water provision, evacuation planning, continuity of care, nutrition support, and context-appropriate vector control and mental health services. The 2026 event illustrates the value of anticipatory action, while these priorities remain relevant across future ENSO cycles.
